# BRD4 Inhibition Enhances the Antitumor Effects of Radiation Therapy in a Murine Breast Cancer Model

**DOI:** 10.3390/ijms241713062

**Published:** 2023-08-22

**Authors:** Seongmin Kim, Seung Hyuck Jeon, Min Guk Han, Mi Hyun Kang, In Ah Kim

**Affiliations:** 1 Department of Tumor Biology, Graduate School of Medicine, Seoul National University, Seoul 03080, Republic of Korea; ksm931204@snu.ac.kr (S.K.); han3270@naver.com (M.G.H.); 2 Integrated Major in Innovative Medical Science, Seoul National University Graduate School, Seoul 03080, Republic of Korea; 3 Medical Science Research Institute, Seoul National University Bundang Hospital, Seongnam-si 13620, Republic of Korea; biokmh@naver.com; 4 Department of Radiation Oncology, Seoul National University Bundang Hospital, 173 Gumiro, Seongnam-si 13620, Republic of Korea

**Keywords:** BRD4, radiation therapy, triple-negative breast cancer, immunosuppressive cells, tumor microenvironment

## Abstract

Bromodomain-containing protein 4 (BRD4) is an intracellular protein that regulates expression of various cellular functions. This study investigated whether BRD4 inhibition can alter the immunomodulatory and antitumor effects of radiation therapy (RT). A murine breast cancer cell line was implanted into BALB/c mice. The dual-tumor model was used to evaluate the abscopal effects of RT. A total of 24 Gy was delivered and BRD4 inhibitor was injected intravenously. Tumor size was measured, and in vivo imaging was performed to evaluate tumor growth. Flow cytometry and immunohistochemistry were performed to examine immunologic changes upon treatment. The combination of BRD4 inhibitor and RT significantly suppressed tumor growth compared to RT alone. BRD4 inhibitor reduced the size of the unirradiated tumor, indicating that it may induce systemic immune responses. The expression of HIF-1α and PD-L1 in the tumor was significantly downregulated by the BRD4 inhibitor. The proportion of M1 tumor-associated macrophages (TAMs) increased, and the proportion of M2 TAMs decreased upon BRD4 inhibition. BRD4 inhibitor expanded CD4^+^ and CD8^+^ T cell populations in the tumor microenvironment. Additionally, splenic monocytic myeloid derived suppressor cells, which were increased by RT, were reduced upon the addition of BRD4 inhibitor. Therefore, the addition of BRD4 inhibitor significantly enhanced the systemic antitumor responses of local RT.

## 1. Introduction

Radiation therapy (RT) is one of the major pillars of cancer therapy and is used to treat a substantial portion of cancers [1], including breast cancer [2]. However, tumors often do not respond to RT or recur after RT [3]; thus, strategies to enhance the antitumor efficacy of RT need to be developed. To date, various systemic therapies have been investigated in clinical trials and preclinical models to improve the efficacy of local RT alone [4]. To discover optimal targets that enhance the antitumor effect of RT, understanding how RT modulates the tumor microenvironment (TME) and cancer cells is crucial [5].

Among numerous mechanisms underpinning the low efficacy of local RT is the induction of immunosuppressive cells in the tumor [6]. Immunosuppressive cells, including tumor-associated macrophages (TAMs), regulatory T cells (Tregs), and myeloid-derived suppressor cells (MDSCs), play crucial roles in regulating antitumor response in the TME [7]. A growing body of evidence suggests that inhibition or depletion of the immunosuppressive cells successfully inhibits the tumor growth and improves antitumor immunity in preclinical models [8,9]. Although RT promotes antitumor immune responses, these immunosuppressive cells increase in the TME after local RT [10,11,12,13], and induction of immunosuppressive cells by RT has been observed in both preclinical models and cancer patients. Several previous studies demonstrated that therapeutic regulation of immunosuppressive cells significantly improved the antitumor effects of local RT [14,15]. Hence, therapeutic strategies that inhibit the induction or expansion of immunosuppressive cells in the TME are expected to enhance the response of the irradiated tumor and improve systemic antitumor immune responses.

**Figure 1:**
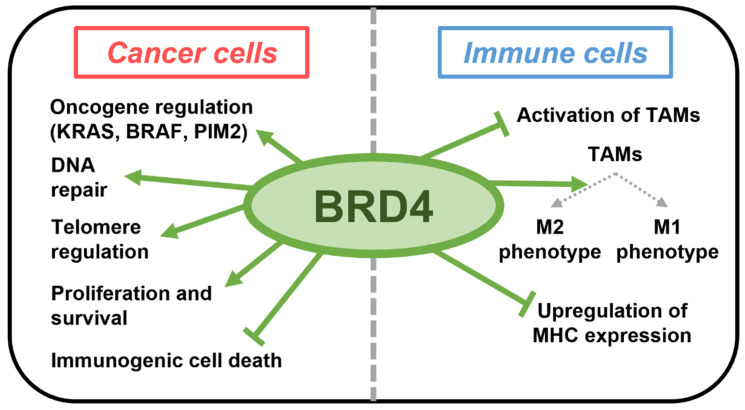
Major roles of BRD4 in cancer and immune cells.

Bromodomain-containing protein 4 (BRD4) is a member of the Bromo- and Extra-Terminal domain (BET) family that is upregulated in tumor cells and regulates gene expression by recruiting various transcription factors by interacting with the acetylated lysine residues of histone tails on chromatins [16]. The target genes of BRD4 include several oncogenes such as KRAS, BRAF, and PIM2. BRD4 is also involved in DNA repair and telomere regulation. Therefore, BRD4 is able to support the proliferation and survival of cancer cells, and targeting BRD4 has shown to be effective in the eradication of tumor cells in preclinical studies [17,18]. In addition to its direct effects on cancer cells, recent evidence suggests that inhibition of BRD4 polarizes TAMs into the M1-like phenotype by inhibiting transcription factors that modulate the phenotype of M2 macrophages including MAF, IRF4, and AKT [19,20]. Inhibition of BRD4 also blocks proliferation of TAMs [21], upregulates expression of MHC molecules [22], and induces immunogenic cell death of tumor cells [23]. These various roles of BRD4 are depicted in Figure 1. Due to these immunologic effects of BRD4 inhibition, a BRD4 inhibitor may improve the antitumor effects of local RT; however, the immunologic effects of combining local RT and BRD4 inhibition have not been explored.

Here, we hypothesized that inhibition of BRD4 is able to enhance the antitumor effects of local RT by inhibiting immunosuppressive cells. We investigated whether BRD4 inhibition improves the antitumor effects of RT in a syngeneic murine triple-negative breast cancer (TNBC) model. We analyzed the immunophenotype of various immune cells, including T cells, TAMs, and MDSCs, according to the administration of BRD4 inhibitor and local RT, and found that the addition of a BRD4 inhibitor to RT led to a less immunosuppressive TME by modulating immunosuppressive cells. Our results suggest that BRD4 may be a viable target to enhance the efficacy of local RT.

## 2. Results

### 2.1. Effects of Local RT and BRD4 Inhibition on Tumor Growth

**Figure 2:**
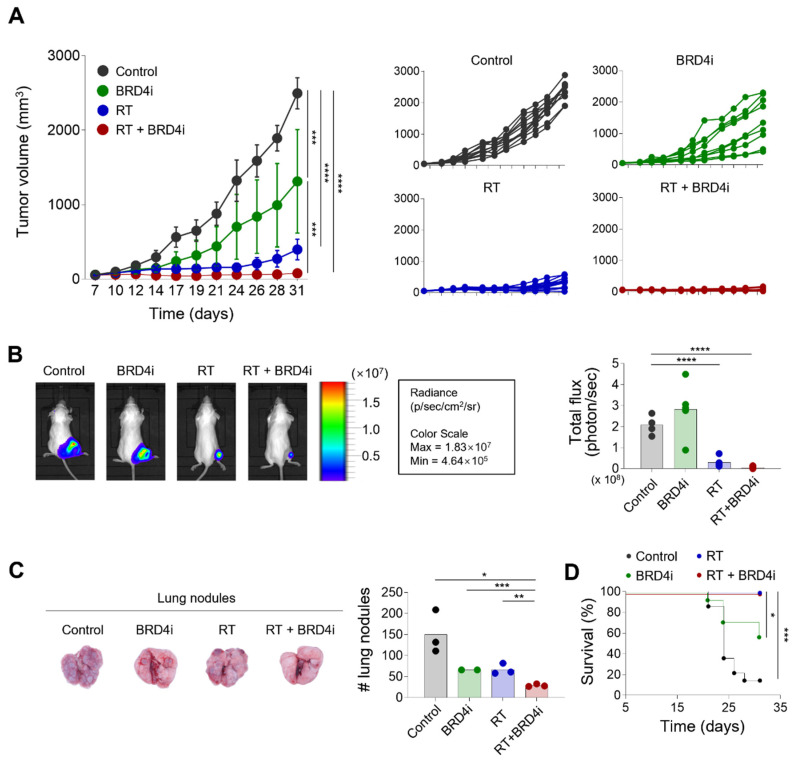
Antitumor effects of radiotherapy (RT) and the BRD4 inhibitor. (**A**) Growth curves of tumors in mice treated with the control, the BRD4 inhibitor, local RT, or combination therapy. (**B**) Representative bioluminescence images (left) and intensities (right) in mice treated with the control, BRD4 inhibitor, local RT, or combination therapy. (**C**) Representative images (left) and numbers of metastatic lung nodules (right) in mice treated with the control, BRD4 inhibitor, local RT, or combination therapy. (**D**) Survival curves of mice treated with the control, the BRD4 inhibitor, local RT, or combination therapy. (****) *p* < 0.0001; (***) *p* < 0.001; (**) *p* < 0.01; (*) *p* < 0.05.

We first examined the antitumor effects of local RT combined with the BRD4 inhibitor. Local RT or BRD4 inhibitor alone delayed tumor growth (Figure 2A). Importantly, combination treatment with local RT and the BRD4 inhibitor reduced tumor growth significantly more than either monotherapy alone. Similarly, in vivo imaging showed that combination treatment resulted in a smaller tumor burden than local RT alone (Figure 2B). Additionally, the numbers of metastatic lung nodules decreased significantly with combination therapy compared to either monotherapy (Figure 2C), suggesting that the BRD4 inhibitor suppresses both local growth and systemic spread of the tumor. Both local RT alone and combination therapy resulted in excellent survival rates for tumor-bearing mice; these rates were significantly better than the survival rates of the control and BRD4 inhibitor-alone mice (Figure 2D). These data indicate that the BRD4 inhibitor enhances the antitumor effects of local RT.

### 2.2. Effects of the BRD4 Inhibitor on the Abscopal Effects of Local RT

**Figure 3:**
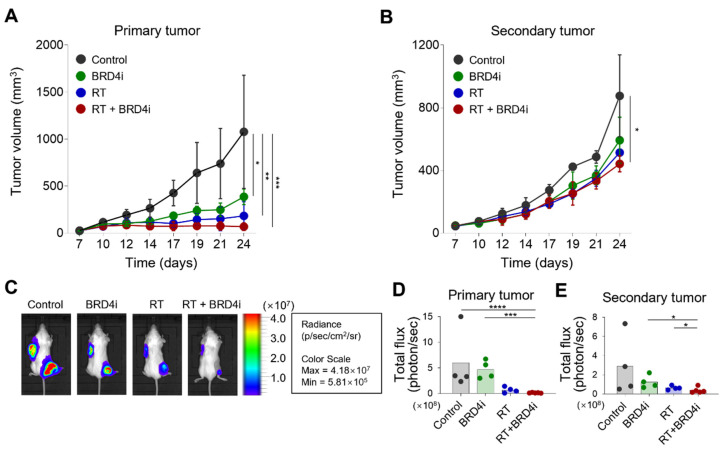
Abscopal effects of RT and the BRD4 inhibitor. (**A**,**B**) Growth curves of primary tumors (**A**) and secondary tumors (**B**) in a dual-tumor model treated with the control, BRD4 inhibitor, local RT, or combination therapy. (**C**) Representative bioluminescence images in the dual-tumor model. (**D**,**E**) Bioluminescence intensities of primary tumors (**D**) and secondary tumors (**E**) in the dual-tumor model treated with the control, BRD4 inhibitor, local RT, or combination therapy. (****) *p* < 0.0001; (***) *p* < 0.001; (**) *p* < 0.01; (*) *p* < 0.05.

To examine whether the addition of the BRD4 inhibitor improves systemic immune responses evoked by local RT, we used the dual-tumor model that was previously used to observe the abscopal effects of RT [24]. RT alone and combination therapy led to similar growth inhibition of the primary tumor (Figure 3A). Notably, combination therapy delayed growth of the secondary tumor when compared to the control, RT-alone, and BRD4-alone groups (Figure 3B). No significant effect on the secondary tumor was observed with RT or BRD4 inhibitor alone. In vivo imaging also demonstrated that combination therapy decreased the burdens of the primary and secondary tumors (Figure 3C–E), suggesting that the BRD4 inhibitor improves the abscopal effects of local RT.

### 2.3. Treatment Effects on the TME

**Figure 4:**
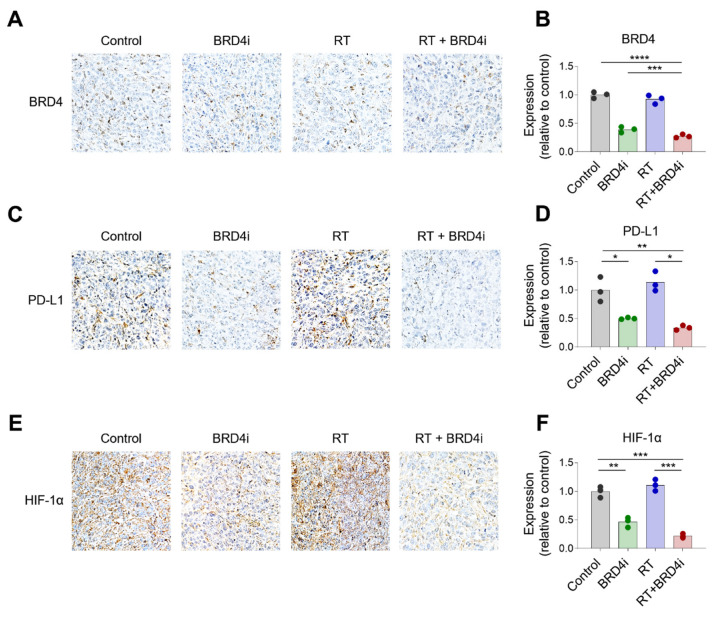
Changes in BRD4, PD-L1, and HIF-1α expression upon RT and BRD4 inhibition. (**A**,**B**) Representative immunohistochemistry (IHC) images (**A**) and relative expression (**B**) of BRD4 in the tissue microenvironment (TME). (**C**,**D**) Representative IHC images (**C**) and relative expression (**D**) of PD-L1 in the TME. (**E**,**F**) Representative IHC images (**E**) and relative expression (**F**) of HIF-1α in the TME. (****) *p* < 0.0001; (***) *p* < 0.001; (**) *p* < 0.01; (*) *p* < 0.05.

Having observed the additional antitumor effects of BRD4 inhibition with local RT, we examined changes in the TME upon local RT and BRD4 inhibition. BRD4 protein expression decreased significantly in the BRD4 inhibitor treatment group, but not in the RT-alone group (Figure 4A,B). Expression of HIF-1α also decreased in the BRD4 inhibitor group (Figure 4C,D), which is consistent with previous reports. Notably, PD-L1 expression was downregulated in the BRD4 inhibitor group (Figure 4E,F), suggesting that BRD4 inhibition may resolve, in part, the immunosuppressive TME.

**Figure 5:**
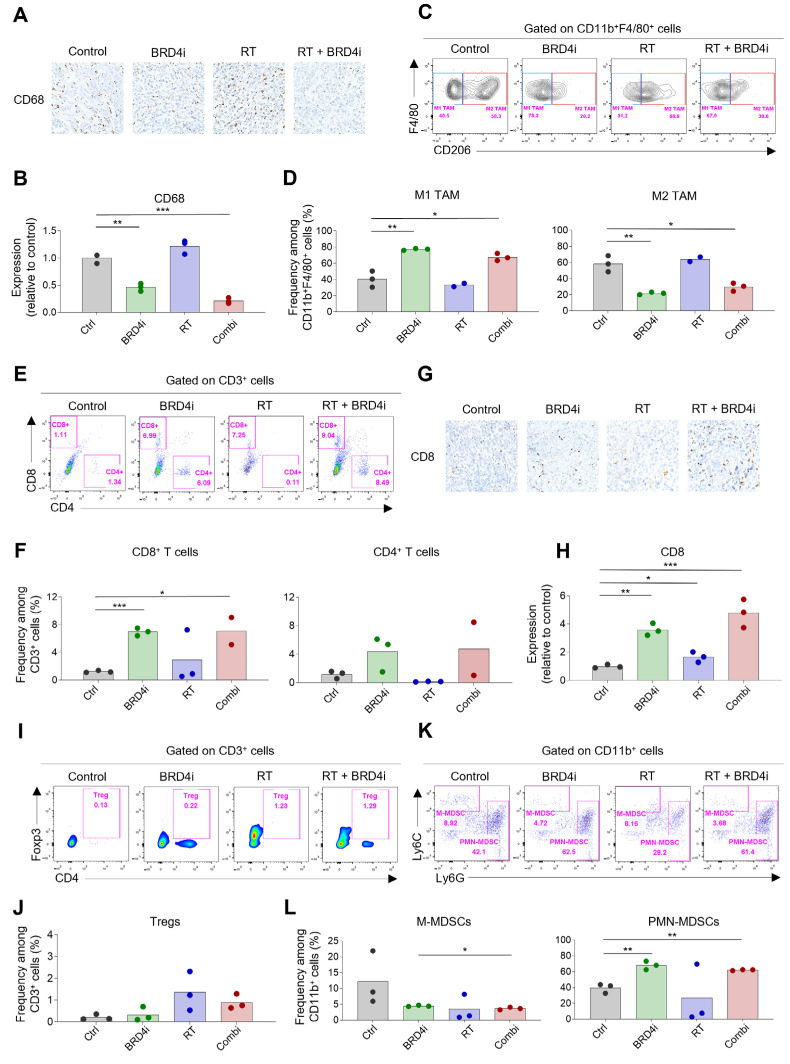
Changes in tumor-infiltrating immune cells by RT and BRD4 inhibition. (**A**,**B**) Representative IHC images (**A**) and relative expression (**B**) of CD68 in tumors. (**C**,**D**) Representative flow cytometry plots (**C**) and frequencies (**D**) of M1 and M2 tumor-associated macrophages (TAMs) in tumors. (**E**,**F**) Representative flow cytometry plots (**E**) and frequencies (**F**) of CD8^+^ T cells and CD4^+^ T cells in tumors. (**G**,**H**) Representative IHC images (**G**) and relative expression (**H**) of CD8 in the TME. (**I**,**J**) Representative flow cytometry plots (**I**) and frequencies (**J**) of regulatory T cells (Tregs) in tumors. (**K**,**L**) Representative flow cytometry plots (**K**) and frequencies (**L**) of monocytic and polymorphonuclear myeloid-derived suppressor cells (MDSCs) in tumors. (***) *p* < 0.001; (**) *p* < 0.01; (*) *p* < 0.05.

Next, we examined the immune cell composition in the TME after treatment. Infiltration of CD68^+^ TAMs decreased upon addition of the BRD4 inhibitor (Figure 5A,B). Treatment with the BRD4 inhibitor significantly increased the proportion of CD206^−^ M1 TAMs and reduced the proportion of CD206^+^ M2 TAMs (Figure 5C,D). Additionally, the BRD4 inhibitor significantly increased the proportion of CD8^+^ T cells (Figure 5E,F). This increase in CD8^+^ T cells was confirmed by IHC (Figure 5G,H). The proportion of Tregs increased upon local RT, and this increase diminished upon the addition of the BRD4 inhibitor; however, the diminution was not statistically significant (Figure 5I,J). We also examined the proportions of MDSCs and found that the frequency of Ly6C^high^ monocytic MDSCs among CD11b^+^ cells decreased upon RT or BRD4 treatment. However, the reductions were not statistically significant. The proportion of Ly6G^high^ polymorphonuclear MDSCs increased upon treatment with the BRD4 inhibitor (Figure 5K,L). These data suggest that combination therapy leads to a more immunogenic TME than local RT alone.

### 2.4. Treatment Effects on Systemic Immune Responses

**Figure 6:**
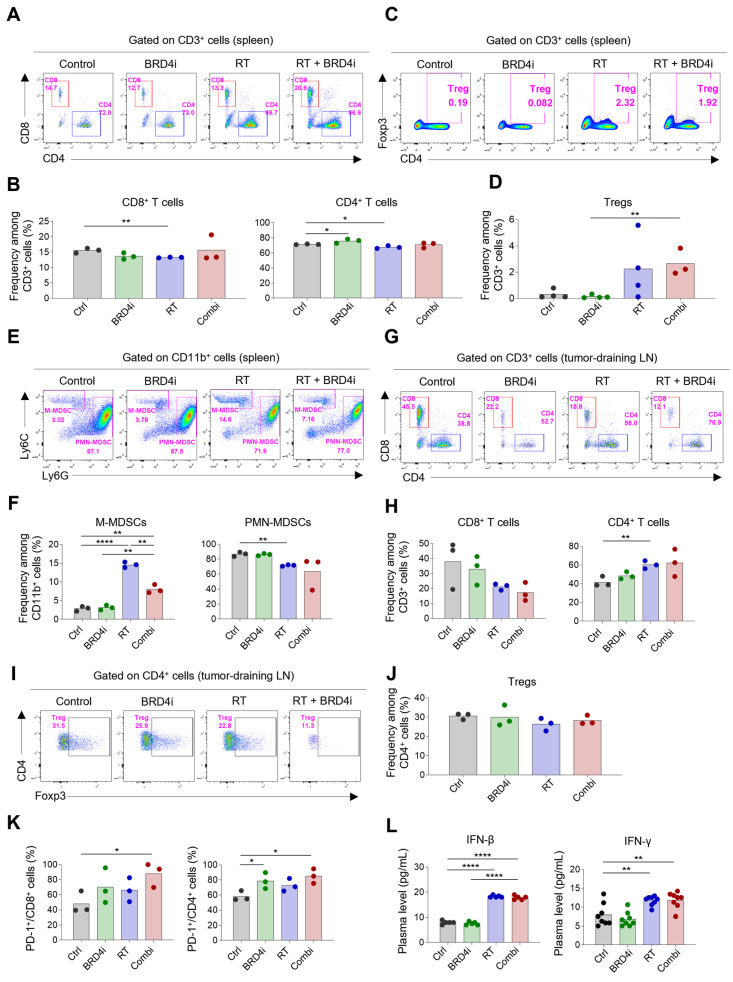
Changes in systemic immune responses by RT and BRD4 inhibition. (**A**,**B**) Representative flow cytometry plots (**A**) and frequencies (**B**) of CD8^+^ T cells and CD4^+^ T cells in spleens. (**C**,**D**) Representative flow cytometry plots (**C**) and frequencies (**D**) of Tregs in spleens. (**E**,**F**) Representative flow cytometry plots (**E**) and frequencies (**F**) of monocytic and polymorphonuclear MDSCs in spleens. (**G**,**H**) Representative flow cytometry plots (**G**) and frequencies (**H**) of CD8^+^ T cells and CD4^+^ T cells in tumor-draining lymph nodes. (**I**,**J**) Representative flow cytometry plots (**I**) and frequencies (**J**) of Tregs in tumor-draining lymph nodes. (**K**) Frequencies of PD-1^+^ cells among CD8^+^ T cells (left) and CD4^+^ T cells in tumor-draining lymph nodes. (**L**) Concentrations of plasma interferon-β (left) and interferon-γ (right). (****) *p* < 0.0001; (**) *p* < 0.01; (*) *p* < 0.05.

Because we observed that the abscopal effects of local RT increased upon the addition of the BRD4 inhibitor, we profiled the immune cells in spleens and the tumor-draining lymph nodes to determine the effects of the treatments on systemic immunity. The proportions of CD8^+^ and CD4^+^ T cells in the spleen were not altered remarkably by any of the treatments (Figure 6A,B), whereas splenic Tregs increased upon combination therapy (Figure 6C,D). Notably, the proportion of monocytic MDSCs increased upon local RT; however, this increase was ameliorated by the addition of the BRD4 inhibitor (Figure 6E,F). The proportion of polymorphonuclear MDSCs was lowest upon combination therapy, although the difference was not statistically significant. In tumor-draining lymph nodes, the proportion of CD4^+^ T cells among total T cells increased upon combination therapy, whereas the proportion of CD8^+^ T cells decreased (Figure 6G,H). Proportions of Tregs did not change significantly upon any of the treatments (Figure 6I,J). Importantly, expression of PD-1 on CD8^+^ and CD4^+^ T cells in tumor-draining lymph nodes increased upon combination therapy (Figure 6K), indicating an abundance of tumor-specific T cells after local RT and BRD4 inhibition. Levels of plasma IFN-β and IFN-γ increased upon RT but were not further elevated upon the addition of the BRD4 inhibitor (Figure 6L). Collectively, these results suggest that BRD4 inhibition may contribute to a systemic decrease in immunosuppressive cells, particularly MDSCs, and an increase in tumor-specific T cells in draining lymph nodes.

## 3. Discussion

The antitumor effect of local RT is often limited by various mechanisms, including induction of an immunosuppressive TME. Discovery of novel strategies to overcome this limitation is necessary to maximize the effects of local RT. In this study, we demonstrated that inhibition of BRD4 enhances the antitumor effects of local RT. BRD4 inhibitor induces an immunostimulatory TME, at least in part, by inhibiting immunosuppressive cells in a breast cancer model. Our study suggests that BRD4 inhibitor may be an optimal combination partner of local RT. To the best of our knowledge, this is the first study to describe immunologic changes by local RT and BRD4 inhibition.

In addition to the direct cytotoxic effect of RT on tumor cells, local RT activates various immunostimulatory and immunosuppressive cells [6]. The immunomodulatory effects of RT have been attributed to various factors including damage-associated molecular patterns, cytokines, upregulation of immune receptors, and release of tumor antigens. Our previous studies using a 4T1-Luc mouse model showed that M2 TAMs, MDSCs, and Tregs increased upon local RT [24,25]. A growing body of evidence suggests that modulating immunosuppressive cells improve the antitumor effect of RT [14,15,26]. Several novel strategies to inhibit immunosuppressive cells to enhance the efficacy of both local RT and immune checkpoint inhibitors have been suggested [24,25]. In this study, we observed that BRD4 inhibition significantly decreased the infiltration of M2 TAMs into the TME. Moreover, circulating M-MDSCs were diminished by the addition of BRD4 inhibitor to local RT. Our findings indicate that BRD4 inhibition can be utilized to enhance the antitumor effect of RT, probably by modulating immunosuppresive cells, including M2 TAMs and MDSCs.

BRD4 is expressed in various cells, including cancer cells and immune cells, and previous investigations on the BRD4 inhibitor focused on its direct effects on cancer cells. Recent studies indicated that BRD4 is also involved in innate immune responses [27,28]. Our finding that the BRD4 inhibitor significantly reduced M2 TAMs within the TME is in accordance with previous studies that showed that BRD4 promoted M2 polarization [19,20]. BRD4 was also shown to be involved in the recruitment of TAMs in a preclinical model [29]. Meanwhile, the role of BRD4 and the effects of BRD4 inhibition on MDSCs have not been elucidated. Since our results demonstrated that splenic M-MDSCs were decreased by adding BRD4 inhibitor to local RT, further research on the role of BRD4 in MDSCs is needed to fully understand the antitumor effects of the BRD4 inhibitor and its ability to improve the efficacy of RT.

The results of this study indicate that the BRD4 inhibitor improves local RT via the reduction of M2 TAMs and M-MDSCs. In addition, the BRD4 inhibitor also regulates the characteristics of tumor cells, because BRD4 is also expressed in tumor cells. Indeed, a previous study showed that BRD4 inhibition directly sensitizes tumor cells to RT by regulating DNA repair [30]. Although we observed remarkable changes in immune cells due to the addition of BRD4 inhibitor, which were well correlated with its antitumor responses, the effects of BRD4 inhibitor in addition to local RT were possibly, at least in part, attributed to its direct effects on cancer cells. Therefore, further studies are needed to dissect the direct and indirect effects of BRD4 inhibition on tumor cells.

In addition to immunologic changes, we observed that the BRD4 inhibitor downregulated HIF-1α expression. Although local RT did not significantly alter HIF-1α expression in our experiment, RT is a known potent inducer of hypoxia, which activates HIF-1α in cancer cells. Hypoxia and HIF-1α have been shown to play various roles in immunity, and hypoxia-driven immunologic changes in the TME tend to inhibit antitumor responses. HIF-1α induces FoxP3, resulting in an abundance of Tregs [31], upregulates PD-L1 that inhibits T cell activation [32], and drives M2-like polarization of TAMs [33]. Hence, downregulation of HIF-1α by BRD4 inhibition may attenuate RT-induced immunosuppression in the TME, as demonstrated in this study.

Recent studies have suggested that tumor-draining lymph nodes play a crucial role in RT-induced antitumor immune responses [34,35]. Priming and activation of tumor-specific T cells take place in tumor-draining lymph nodes and the effects of immune checkpoint inhibitors are regulated in lymph nodes [36]. Tumor-specific T cells that egressed from lymph nodes into the circulation can be recruited into the TME; therefore, activation of systemic immune cells is as important as activation of tumor-infiltrating immune cells. Although we did not observe significant effects on immune cells in tumor-draining lymph nodes upon RT or BRD4 inhibition, we noticed a remarkable increase in splenic T cells. Therefore, BRD4 inhibition may mitigate the immunosuppressive TME and stimulate systemic antitumor immune responses, which should be investigated further in future studies.

This study employed a murine TNBC model, which showed clear abscopal effects in our previous studies [24,25]. Breast cancer is considered a less immunogenic tumor; thus, it is believed that immunotherapy provides limited clinical benefit [37]. However, among the subtypes of breast cancer, TNBC is relatively immunogenic, with a high tumor mutational burden; thus, immunotherapy provides significant benefits [38,39]. Recent evidence from single-cell profiling suggests that the characteristics and constitution of tumor-infiltrating myeloid cells differ substantially according to cancer type [40]. Because our data demonstrated that the antitumor effects of the BRD4 inhibitor were accompanied by alterations in TAMs and MDSCs within the TME, the immunomodulatory effects of the BRD4 inhibitor may vary across tumor types. Future studies should be performed to expand our findings to other cancer types.

Our analysis supports the effectiveness of BRD4 inhibitor in treating cancer, especially breast cancer. Anti-PD-1 blocking antibody is currently used in patients with TNBC [38,39]. In addition, several immunotherapeutic antibodies are under active investigation in various types of cancer [41]. Unlike most immune checkpoint receptors that are expressed on the cell surface, BRD4 is an intracellular target. Therefore, small molecule inhibitors that are orally available can be developed, which, in turn, facilitates administration to cancer patients. Indeed, several clinical trials to examine the efficacy of BRD4 inhibitors are ongoing [42]. Notably, a recent clinical study demonstrated that local RT significantly improved the clinical outcomes of patients undergoing immune checkpoint blockade [43]. In our study, the combination treatment significantly delayed the growth of an unirradiated secondary tumor, compared to BRD4 inhibitor alone. Therefore, the addition of local RT can be considered in future studies of BRD4 inhibitors based on the findings from our experiments.

In summary, the BRD4 inhibitor enhanced the antitumor effects of RT, including its abscopal effects. The BRD4 inhibitor increased antitumor immune responses in the TME, specifically by decreasing the proportion of M2 TAMs and expression of PD-L1. It also reduced splenic M-MDSCs when combined with RT. Therefore, the BRD4 inhibitor presumably synergizes with local RT by modulating immunosuppression. Clinical studies using the BRD4 inhibitor are warranted to validate its benefit in combination with RT.

## 4. Materials and Methods

### 4.1. Cell Culture

The luciferase-tagged 4T1 (4T1-Luc) murine TNBC cell line was purchased from the Japanese Collection of Research Bioresources Cell Bank (Osaka, Japan). The cell line was maintained in complete RPMI1640 medium supplemented with 10% fetal bovine serum and 1% penicillin/streptomycin. The cells were maintained at 37 °C in a 5% CO_2_ incubator.

### 4.2. Mouse Tumor Model

All animal experiments were conducted using immune-competent BALB/c female mice (7 weeks of age) from Orient Bio Inc. (Seongnam, Korea). The 4T1-Luc cells (6 × 10^5^ cells) were subcutaneously injected into the right hindlimb. For the dual-tumor model, the 4T1-Luc cells were subcutaneously injected into the right hindlimb (6 × 10^5^ cells) and left flank (10^5^ cells). Seven days after tumor injection, mice were allocated into 4 treatment groups: control, RT alone, BRD4 inhibitor alone, and combination treatment. All experiments were performed in compliance with animal ethics rules and in accordance with the IACUC Animal Experiment Protocol (BA-2103-316-021-01) of Seoul National University Bundang Hospital.

### 4.3. Treatments

BRD4 inhibitor (OPT-0139; 10 mg/kg) was injected intravenously on days 10, 12, 14, 17, 19, and 21 post-inoculation. RT was delivered on days 10, 12, and 14 post-inoculation using X-Rad320 irradiator (Precision X-Ray Inc, North Branford, CT, USA); a 6 MeV electron beam was used to administer 8 Gy per treatment, leading to irradiation with a total of 24 Gy with a calibrated source-to-specimen distance of 70 cm. For irradiation, mice were anesthetized with intraperitoneal injection of 40% alfaxalone and 20% xylazine. The field size was adjusted to the size of primary tumor to avoid irradiation to secondary tumor. On the 31st day after tumor implantation, mice were sacrificed and lungs, draining lymph nodes, spleens, and tumor tissues were harvested. Half of an extracted tumor was fixed in 4% paraformaldehyde and embedded in paraffin for immunohistochemistry (IHC). The obtained lymph nodes, spleens, and tumors were separated into single cells and stored at −70 °C for subsequent flow cytometry analysis.

### 4.4. Tumor Growth

Tumor size was measured using a caliper, and tumor volume was calculated as Volume = (*D* × *d*^2^)/2, in which D and d represent the long and short diameters, respectively. Luciferase solution was used to obtain bioluminescence images, and the images were analyzed using the IVIS Lumina III In Vivo Imaging System (PerkinElmer, Waltham, MA, USA) on day 31 post-inoculation.

### 4.5. Immunohistochemistry (IHC)

Tumor tissues embedded in paraffin blocks were cut into 4 μm-thick transverse slices and attached to glass slides. Tissue slices were deparaffinized using xylene and ethanol and then immersed in a solution of 3% H_2_O_2_ in methanol at room temperature for 10 min. Then, tissue slices were boiled in 0.01 M sodium citrate buffer pH 6.0 and blocked with 5% normal goat serum. Tissue slices were incubated with primary antibodies targeting BRD4, HIF-1α, PD-L1, CD8, and CD68 overnight at 4 °C. Images were collected using an Axioskop 40 light microscope (Carl Zeiss, Jena, Germany) and AxioVision 4.7 software. Image J software V1.52 was used for quantification, and the average density value was calculated for at least 3 slides per sample.

### 4.6. Flow Cytometric Analysis

After thawing, cells were treated with Fc blocking agent (Biolegend: 156604, San Diego, CA, USA) at 4 °C for 15 min and incubated with fluorochrome-conjugated antibodies against the surface markers at 4 °C for 30 min. Then, cells were fixed and permeabilized using the Foxp3 Buffer Set (BD Biosciences: 560098, San Diego, CA, USA) according to the manufacturer’s instructions. After washing, cells were incubated with fluorochrome-conjugated antibodies against Foxp3 at 4 °C for 30 min. Flow cytometry analysis was performed using FACSCalibur (BD Biosciences) and FACSDiva software Version 6.1.3 (BD Biosciences). All data were analyzed using FlowJo software Version 10 (BD Biosciences). All primary antibodies used for flow cytometry are listed in Supplementary Table S1.

### 4.7. Statistical Analysis

Statistical analyses were performed using GraphPad PRISM V8.0.2 (GraphPad Software Inc., San Diego, CA, USA). Two-way ANOVA was used to compare tumor growth in the mouse tumor model. The unpaired Student’s *t*-test was used to compare continuous variables between two groups. Significance was defined as a *p* value < 0.05.

## Data Availability

Not applicable.

## Supplementary Materials

The following supporting information can be downloaded at: https://www.mdpi.com/article/10.3390/ijms241713062/s1.
