# Correction: Kim et al. BRD4 Inhibition Enhances the Antitumor Effects of Radiation Therapy in a Murine Breast Cancer Model. *Int. J. Mol. Sci.* 2023, *24*, 13062

**DOI:** 10.3390/ijms262110413

**Published:** 2025-10-27

**Authors:** Seongmin Kim, Seung Hyuck Jeon, Min Guk Han, Mi Hyun Kang, In Ah Kim

**Affiliations:** 1 Department of Tumor Biology, Graduate School of Medicine, Seoul National University, Seoul 03080, Republic of Korea; ksm931204@snu.ac.kr (S.K.); han3270@naver.com (M.G.H.); 2 Integrated Major in Innovative Medical Science, Seoul National University Graduate School, Seoul 03080, Republic of Korea; 3 Medical Science Research Institute, Seoul National University Bundang Hospital, Seongnam-si 13620, Republic of Korea; biokmh@naver.com; 4 Department of Radiation Oncology, Seoul National University Bundang Hospital, 173 Gumiro, Seongnam-si 13620, Republic of Korea; hyck9004@naver.com

In the original publication [1], there was a mistake in the left graph of Figure 5L as published. We inadvertently duplicated the graph for PMN-MDSCs and used it in place of the graph intended to represent M-MDSCs. As a result, both graphs in Figure 5L displayed the same PMN-MDSC data, and the actual M-MDSC data was omitted.

The corrected Figure 5L appears below. The authors state that the scientific conclusions are unaffected. This correction was approved by the Academic Editor. The original publication has also been updated.

## Figures and Tables

**Figure 5:**
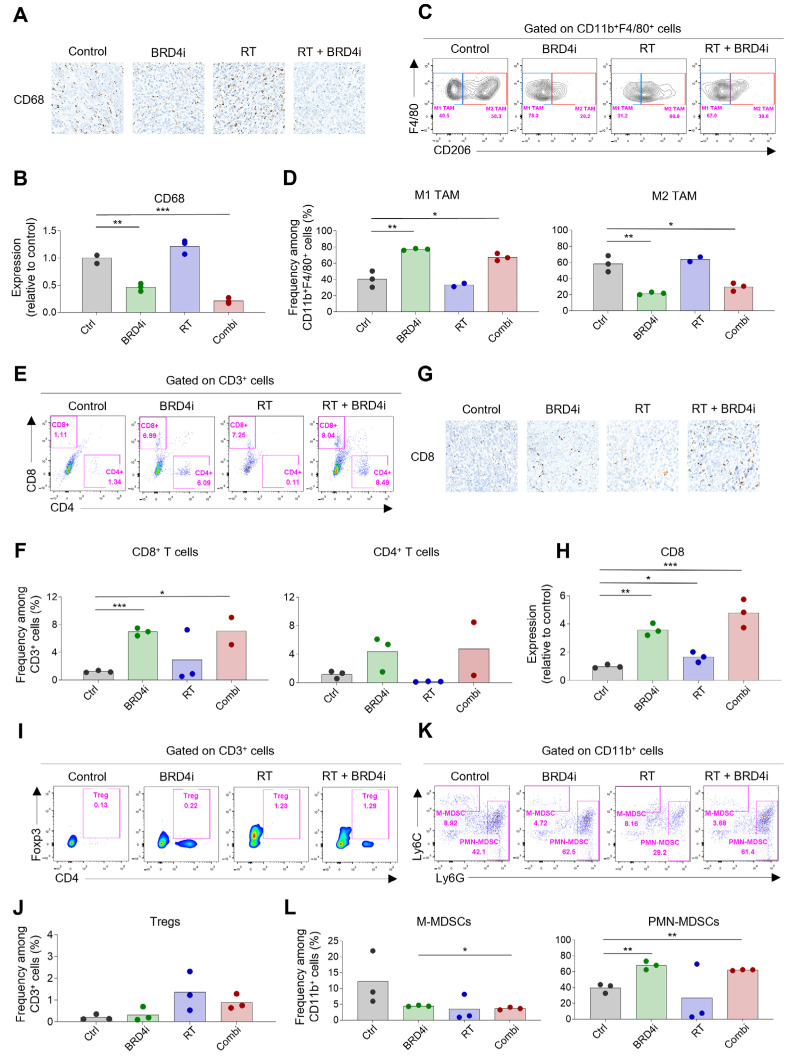
Changes in tumor-infiltrating immune cells by RT and BRD4 inhibition. (**A**,**B**) Representative IHC images (**A**) and relative expression (**B**) of CD68 in tumors. (**C**,**D**) Representative flow cytometry plots (**C**) and frequencies (**D**) of M1 and M2 tumor-associated macrophages (TAMs) in tumors. (**E**,**F**) Representative flow cytometry plots (**E**) and frequencies (**F**) of CD8^+^ T cells and CD4^+^ T cells in tumors. (**G**,**H**) Representative IHC images (**G**) and relative expression (**H**) of CD8 in the TME. (**I**,**J**) Representative flow cytometry plots (**I**) and frequencies (**J**) of regulatory T cells (Tregs) in tumors. (**K**,**L**) Representative flow cytometry plots (**K**) and frequencies (**L**) of monocytic and polymorphonuclear myeloid-derived suppressor cells (MDSCs) in tumors. (***) *p* < 0.001; (**) *p* < 0.01; (*) *p* < 0.05.

## References

[1] Kim S., Jeon S.H., Han M.G., Kang M.H., Kim I.A. (2023). BRD4 Inhibition Enhances the Antitumor Effects of Radiation Therapy in a Murine Breast Cancer Model. Int. J. Mol. Sci..

