# Supplementary material for: BRD4 Inhibition Enhances the Antitumor Effects of Radiation Therapy in a Murine Breast Cancer Model
Source: Int J Mol Sci. 2023 Aug 22;24(17):13062. doi: 10.3390/ijms241713062 (PMC10487493; doi:10.3390/ijms241713062)
Supplement: Supplementary file 1 [file ijms-24-13062-s001.zip › ijms-2549342-supplementary.pdf]

Table S1. List of flow cytometric antibodies used in the study.

Reagent	Clone	Source	Identifier
FITC Rat Anti-Mouse CD3	17A2	Biolegend	#100204
FITC Rat Anti-Mouse/Human CD11b	M1/70	Biolegend	#101206
FITC Rat Anti-Mouse CD25	PC61	Biolegend	#102005
FITC Rat Anti-Mouse CD45	30-F11	BD Biosciences	#553080
PE Rat Anti-Mouse CD8a	53-6.7	Biolegend	#100708
PE Rat Anti-Mouse Foxp3	NRRF-30	Invitrogen	#12-4771-82
PE Rat Anti-Mouse F4/80	T45-2342	BD Biosciences	#565410
PE Rat Anti-Mouse Ly6G	1A8	Biolegend	#127607
APC Rat Anti-Mouse CD4	RM4-5	Biolegend	#100516
APC Rat Anti-Mouse/Human CD11b	M1/70	Biolegend	#101212
APC Rat Anti-Mouse CD206	C068C2	Biolegend	#141708
PerCP-Cy5.5 Rat Anti-Mouse CD3	17A2	Biolegend	#100218
PerCP-Cy5.5 Rat Anti-Mouse Ly6C	HK1.4	Biolegend	#128011
PerCP-Cy5.5 Mouse Anti-Human/Mouse Granzyme B	QA16A02	Biolegend	#372211
